# Non‐Invaginated Wing Primordia in Holometabolous Insects: First Report From Mecoptera

**DOI:** 10.1111/ede.70048

**Published:** 2026-06-07

**Authors:** Shuhei Niitsu, Ryo Ishihara, Takehiko Kamito

**Affiliations:** ^1^ Department of Biological Sciences Tokyo Metropolitan University Hachioji Tokyo Japan; ^2^ Department of Arts and Sciences International Christian University Mitaka Tokyo Japan; ^3^ The University Museum The University of Tokyo Bunkyo‐ku Tokyo Japan; ^4^ Department of Biology, Faculty of Science Shinshu University Matsumoto Nagano Japan

**Keywords:** Holometabola, Mecoptera, metamorphosis, non‐invaginated wing primordia

## Abstract

This study presents the first detailed histological description of wing primordium development in Mecoptera and demonstrates a late‐forming, non‐invaginated developmental mode lacking a morphologically distinct peripodial epithelium. Wing primordium development in holometabolous insects follows two major modes, early‐forming and late‐forming. Late‐forming primordia are epidermal cell populations that remain integrated within the larval epithelium, contribute to cuticle production during each larval molt, and begin morphogenesis only in the final instar. The order Mecoptera remains among the least studied groups of Holometabola with respect to wing primordium development. Using a newly established rearing method and histological analysis, we investigated wing development in the scorpionfly *Mavropanorpa japonica* and found the following: (1) wing primordia are absent in actively feeding larvae; (2) they originate as localized epidermal thickenings beneath the lateral pinacula approximately 20 days after cocoon formation; (3) thickening of wing primordia begins concurrently with apolysis, and the epithelium extends outward without pouch‐like invagination; and (4) no morphologically distinct peripodial epithelium was observed at all stages. Our findings show that wing primordia in Mecoptera develop externally, without invagination and without formation of a morphologically distinct peripodial epithelium. This study provides new comparative insight into heterochronic shifts and evolutionary diversity in holometabolous wing development.

## Summary

We provide the first detailed histological description of a late‐forming, non‐invaginated wing primordium lacking a morphologically distinct peripodial epithelium in Mecoptera.

This study provides new comparative insight into heterochronic shifts and evolutionary diversity in holometabolous wing development.

## Graphical abstract

We investigated wing primordium development in the scorpionfly *Mavropanorpa japonica* and present the first detailed histological description of a late‐forming, non‐invaginated wing primordium lacking a morphologically distinct peripodial epithelium in Mecoptera. The schematic contrasts this externally developing condition with the early‐forming, invaginated mode characteristic of many holometabolous insects.

## 1 Introduction

Holometabolous insects (Endopterygota) develop their wing primordia within the integument and lack external wing projections during the larval stage, although pupal wing pads are generally recognizable. The patterns of wing primordium development have been described since the early 20th century (Mercer 1900; Tower 1903; Powell 1905; Tannreuther 1910; Köhler 1932), and two broadly recognized developmental modes have been observed in Holometabola: early‐forming and late‐forming wing primordia. Early‐forming wing primordia invaginate during embryogenesis or early larval instars and become morphologically segregated from the outer larval epidermis, although they remain continuous with it through the peripodial layer, whereas late‐forming wing primordia remain integrated within the larval epidermis and initiate morphogenesis only during the final instar. The late‐forming condition has often been interpreted as the ancestral state of wing primordium development in Holometabola, based on both comparative morphology and endocrinology (Truman and Riddiford 1999, 2002).

S̆vácha (1992) critically re‐evaluated the classical concept of imaginal discs and demonstrated that epidermal continuity and larval cuticle production are fundamental features of holometabolous imaginal primordia. In this framework, invaginated, early‐forming discs such as those of higher Diptera and some Lepidoptera are interpreted as derived conditions. Late‐forming primordia, by contrast, retain direct continuity with the larval epidermis, contribute to successive larval cuticles, and only later cease cuticle secretion, undergo apolysis, and enter a proliferative morphogenetic phase. This mode of development closely resembles the postembryonic growth pattern of epidermal appendage primordia in hemimetabolous insects, supporting the interpretation of this developmental mode as plesiomorphic within Holometabola (S̆vácha 1992). This interpretation is further supported by endocrine and developmental evidence reviewed by Truman (2019). In the basal holometabolous developmental program, wing primordia remain under juvenile hormone (JH) control during preterminal larval instars, maintaining a proportional growth mode tightly coupled to larval molting cycles. Even during the final larval instar, these primordia continue to function as part of the larval epidermis and contribute to external larval cuticle formation until the onset of morphogenesis. Only in the final larval instar, when JH declines and ecdysteroid signaling is activated, do these primordia enter morphogenesis. According to this model, late‐forming wing primordia represent the ancestral condition in which adult structures arise through delayed activation of embryonic growth programs, whereas early‐forming, deeply invaginated discs reflect a derived acceleration and spatial segregation of morphogenesis from the larval epidermis.

S̆vácha (1992) further noted that he was unable to detect wing discs in *Panorpa* or other mecopteran taxa, suggesting a late‐forming developmental mode in Mecoptera. However, phylogenetic relationships within broadly defined Mecoptera remain unsettled. The group has traditionally been divided into Pistillifera (including Panorpidae), Boreidae, Nannochoristidae, and Siphonaptera, and recent molecular analyses often recover Nannochoristidae as sister to Siphonaptera rather than as the basal lineage of Mecoptera. Notably, Kristensen (1999) reported deeply invaginated wing rudiments enclosed within a peripodial sheath in *Nannochorista*, indicating that wing primordium morphology may vary substantially among mecopteran lineages. Among extant Mecoptera, *Panorpa* (Panorpidae) is considered one of the more derived lineages within the order, yet their wing development has never been histologically documented. This delayed developmental mode has also been observed in several holometabolous lineages, including Megaloptera, Raphidioptera, Mecoptera, and certain groups of Diptera, Coleoptera, and Hymenoptera (Symphyta) (S̆vácha 1992; Sehnal et al. 1996; Truman and Riddiford 1999, 2002). Therefore, comparative ontogenetic investigations of late‐forming wing primordia are essential for understanding the ancestral state of holometabolous wing development. Despite the evolutionary significance of this developmental pattern, ontogenetic studies remain scarce, with the exceptions of early observations by Weismann (1866), Tower (1903), and Quennedey and Quennedey (1990). A major limitation in this field has been the difficulty in rearing most holometabolous species other than established insect models, which has hindered direct observation of their developmental processes. Consequently, many aspects of wing primordium formation in Raphidioptera, Megaloptera, Mecoptera, and certain lineages of Diptera, Coleoptera, and Hymenoptera (Symphyta) remain poorly understood.

Rottmar (1966) reported the postembryonic development of compound eyes during the larval‐pupal period and noted the location of wing primordia in the meso‐ and metathorax of *Panorpa communis*. However, wing primordium morphogenesis was not described in detail. The precise timing and morphological transitions during wing primordium development in Mecoptera have not yet been described in detail. A major limitation has been the limited knowledge of larval life histories and rearing methods in this order compared with other holometabolous insect groups.

In this study, we established an individual larval rearing method based on the protocol of Ishihara and Miyatake (2022), which allowed reliable staging after cocoon formation. This methodological advancement enabled detailed histological observation of the entire processes of wing primordium development in Mecoptera, particularly during the prepupal stage. To elucidate the ontogenetic process of wing primordium development in Eumecoptera, we investigated the wing primordia of the scorpionfly *Mavropanorpa japonica*. Based on our findings, we discuss the evolutionary implications of this developmental mode and its relevance to the evolutionary origin of wing primordia in Holometabola.

## 2 Materials and Methods

### 2.1 Rearing and Staging

Two males and eight females of *Mavropanorpa japonica* were collected in Hachioji City, Tokyo, Japan in 2023. Mass rearing of larvae was conducted following the method of Ishihara and Miyatake (2022). Hatched larvae were maintained under a photoperiod of 16 L:8D at 20°C in an incubator. Newly ecdysed final‐instar larvae were removed from mass culture and reared individually in plastic Petri dishes (35 mm in diameter) containing thawed crickets, with the dishes lined with moistened filter paper. Larvae that ceased feeding and began wandering were transferred to a new plastic Petri dish (6 cm in diameter) containing moist soil and dead leaves, where they formed soil cocoons at the bottom of the dish approximately 4–5 days after transfer.

Samples for histological analysis were collected from actively feeding penultimate‐ and final‐instar larvae. Preliminary rearing experiments were conducted under controlled conditions (20°C, 16 L:8D photoperiod). The duration from cocoon formation to pupation was approximately 40 days. Following cocoon formation, histological samples were collected at 5‐day intervals until pupation.

### 2.2 Identification of Wing Primordia and Histological Analysis

To determine the precise position of the wing primordia, serial semi‐thin sections of the thoracic region were prepared. The sections were referenced against chaetotaxy patterns described by Cai and Hua (2009) to ensure accurate identification.

Larvae were dissected by making transverse incisions across the thoracic region under a stereomicroscope. Tissues, including wing primordia, were fixed in Karnovsky's fixative (2% paraformaldehyde and 2.5% glutaraldehyde) to preserve ultrastructural integrity, followed by postfixation in 1% osmium tetroxide to enhance membrane contrast. After dehydration through a graded ethanol series and propylene oxide, the tissues were embedded in Epon 812 (TAAB). Semi‐thin sections (1 μm thick) were prepared and stained with Azure B before observation under a light microscope (UC‐7; Leica, Germany).

**Figure 1:**
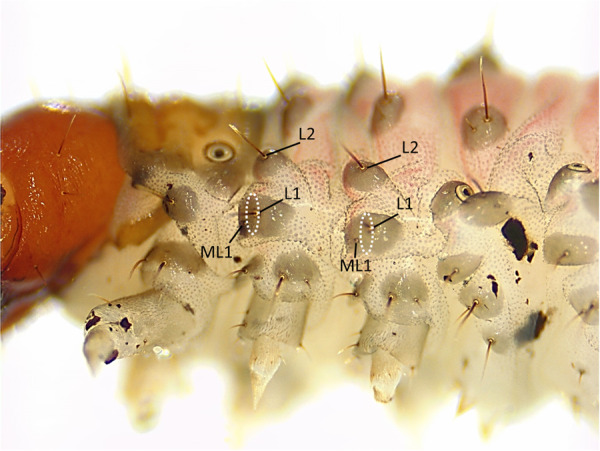
*Mavropanorpa japonica*. Lateral view of the final larval instar. The white dotted line indicates the approximate position of the future wing primordia beneath the L1 setae on the meso‐ and metathorax. [Color figure can be viewed at wileyonlinelibrary.com]

For histological observation, cross‐sections were made perpendicular to the epidermal surface, passing through the region of the L1 and L2 setae on the mesothorax (Figure 1). A total of three larvae per developmental stage were examined to ensure consistency. Observations focused on the anterior view of the mesothoracic wing primordium. When necessary, wing primordium orientation was standardized to the left side using Adobe Photoshop Elements 2019. To ensure consistent identification of the same anatomical structures across developmental stages, we followed the chaetotaxy terminology for the mesothorax described by Cai and Hua (2009). Precise localization of the wing primordia was achieved through the preparation of 1 μm semi‐thin sections, stained with Azure B and cross‐referenced against chaetotaxy patterns.

## 3 Results

**Figure 2:**
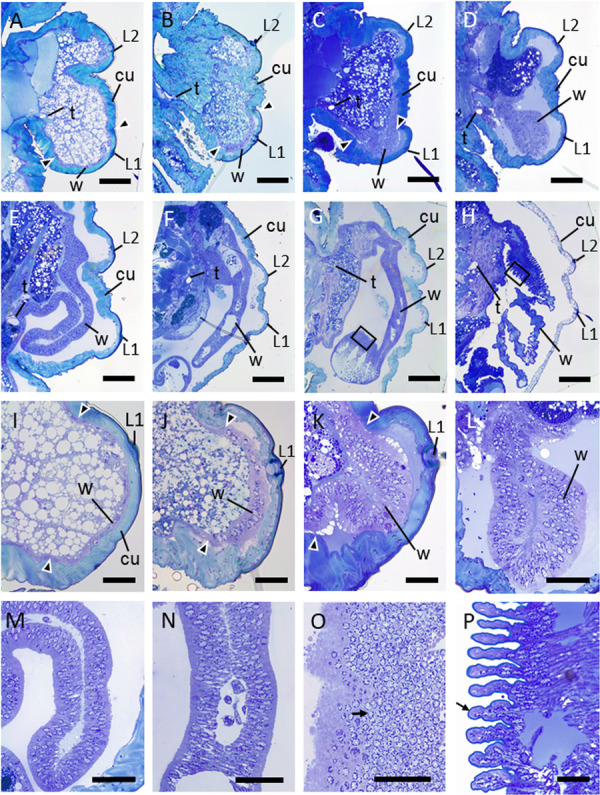
Cross‐sections through the mesothoracic wing primordium in *Mavropanorpa japonica*. Actively feeding final instar (A and I), day 20 after cocoon formation (B and J), day 25 (early stage) (C and K), day 25 (late stage) (D and L), day 30 (early stage) (E and M), day 30 (late stage) (F and N), day 35 (early stage) (G and O), and day 35 (late stage) (H and P). The dorsal surface is at the top in (A–H). The region between the two arrowheads indicates the site of initial apolysis and epithelial thickening (B–C, J–K), marking the region from which the wing primordium subsequently develops (A and I). No pouch‐like invagination of the wing primordium into the body cavity was observed. Note the absence of a morphologically distinct peripodial epithelium. Tracheae lie slightly medial to the primordium rather than at its base (A–H). Arrow in (O) indicates densely packed nuclei in the wing blade. Arrow in (P) indicates pupal cuticle secretion on the developing pupal wing surface. w, wing epithelium; cu, cuticle; t, trachea. Scale bars: 100 μm in A–E, 200 μm in F–H, and 50 μm in I–P. [Color figure can be viewed at wileyonlinelibrary.com]

In actively feeding final‐instar larvae and in individuals at day 15 after cocoon formation, the larval epithelium in the presumptive wing region remained flat and continuous with the surrounding epidermis, showing no evidence of thickening or structural differentiation (Figure 2A,I). A tracheal branch was located slightly medial to the primordium epithelium (Figure 2A), suggesting early spatial association with internal tissues. Similar tracheal positioning was also observed from day 20 through day 35. No wing primordia were detectable beneath the larval cuticle in the lateral meso‐ and metathoracic regions (Figure 2A,I).

At day 20 after cocoon formation (Figure 2B,J), parts of the larval epidermis began to detach from the cuticle, indicating partial apolysis, and a localized cylindrical thickening formed (Figure 2J), indicating the initial emergence of a distinct tissue domain. At the early stage of day 25 after cocoon formation (Figure 2C,K), the epithelial monolayer of the wing primordium proliferated and began to extend slightly toward the body lumen without forming an invaginated structure (Figure 2K).

At the late stage of day 25 after cocoon formation (Figure 2D,L), the wing primordium extended directly toward the ventral inner surface of the cuticle. Apolysis was partially evident beneath the L1 and L2 setae. As the wing primordium elongated, the region within the inner cavity of the epidermis, presumably the fat body, gradually regressed, and the wing primordium expanded into this space (Figure 2L). No morphologically distinct peripodial epithelium was detectable in serial sections at any examined stage (Figure 2). Mitotic activity was consistently observed from day 25 onward, indicating progressive tissue proliferation (Figure 2L–N).

At the early stage of day 30 after cocoon formation (Figure 2E,M), the wing primordium continued to elongate and adopt an l‐shaped conformation. The epidermal pocket region ventral to the L1 setae appeared loosened and expanded. The lumen of the wing primordium became more distinct and expanded (Figure 2E), and mitotic figures were frequently observed. The inner region of the wing primordium became clearly differentiated into two epithelial layers, which could be readily distinguished (Figure 2M).

At the late stage of day 30 after cocoon formation (Figure 2F,N), the wing primordium extended further ventrally. Apolysis expanded further around the wing primordium, and the primordium became increasingly slender as it elongated ventrally. Within the lumen of the wing primordium, pocket‐like spaces began to form (Figure 2N).

In the early stage of day 35 after cocoon formation (Figure 2G,O), the wing primordium continued to elongate ventrally. The distal tip of the wing primordium differentiated into a rounded, bulbous structure. High‐magnification images revealed densely packed nuclei in this region, suggesting intensive cellular activity (Figure 2O).

At the late stage of day 35 after cocoon formation (Figure 2H,P), the wing primordium began to fold, and a new pupal cuticle was secreted over its surface, marking the onset of pupal transition (Figure 2P).

## 4 Discussion

Our histological observations revealed that the wing primordium of the scorpionfly *Mavropanorpa japonica* originates as a thickened epidermis without undergoing the typical epidermal invagination of early‐forming discs. Specifically, our results show that the wing primordium arises from epidermal cells located beneath the lateral pinacula of the L1 setae, first appearing as localized thickenings around 20 days after cocoon formation. Unlike the invagination process characteristic of early‐forming wing discs in many holometabolous insects, the wing epithelium in *M. japonica* thickens and progressively extends toward the ventral inner surface of the cuticle without forming a pouch‐like structure. Apolysis and gradual regression of underlying tissues, including fat body, permit primordium expansion without invagination. The frequent mitoses observed from day 25 onward indicate active cell proliferation during the transition from primordium formation to elongation. The most significant findings of this study are that apolysis begins concurrently with localized thickening in the wing primordium region and that no morphologically distinct peripodial epithelium is formed. This synchrony suggests a direct developmental shift from a cuticle‐producing epidermis to a morphogenetically active disc epithelium. Primordium formation therefore appears to initiate during the prepupal stage, representing a temporal delay in morphogenesis relative to early‐forming discs. In most other holometabolous insects, tracheae are located at the base of the wing primordia from the earliest stages of morphogenesis. In contrast, in *M. japonica*, we found that the trachea was located slightly apart from the epithelium of the wing primordium rather than directly at its base. This positioning highlights a previously unrecognized feature of non‐invaginated primordium morphogenesis and warrants further investigation in future comparative studies.

Tower (1903) described a “simple type” of wing disc in certain Coleoptera that developed externally without deep invagination. S̆vácha (1992) later cited this observation and suggested that epidermally continuous, non‐invaginated primordia may reflect the ancestral condition of Holometabola. Whether the non‐invaginated mode is plesiomorphic or represents a secondary reduction remains unresolved. The phylogenetic distribution of disc morphologies is still incompletely understood, and broader comparative developmental analyses are required to determine the evolutionary polarity of wing disc formation.

Serial semi‐thin sections revealed that the wing primordium of *M. japonica* remains continuous with the surrounding epidermis throughout development. Accordingly, no morphologically distinct peripodial epithelium was observed at any stage. Although invaginated wing discs remain continuous with the larval external epidermis through the cellular peripodial epithelium, our observations indicate that a morphologically distinct peripodial layer is not a universal feature of holometabolous wing development and may be specifically associated with deeply invaginated developmental modes.

Several studies have indicated that holometabolous insects lacking larval wing discs often develop wings externally, without invagination, during metamorphosis (S̆vácha 1992), and intermediate developmental modes between external and internal discs have been reported in *Tenebrio molitor* (Coleoptera) (Quennedey and Quennedey 1990). In the present study, the observed mode of formation is clearly external and lacks any sign of invagination. Our findings provide the first continuous, stage‐specific account of non‐invaginated, externally developing wing primordia in a holometabolous lineage, representing a developmental mode that has not been previously documented in detail.

Our findings also provide new insights into the evolutionary interpretation of wing primordium development within Mecoptera. Kristensen (1999) reported that in the active final instar of *Nannochorista* (Mecoptera), the wing disc is completely enclosed within a peripodial sheath. Based on this observation, Kristensen tentatively proposed that invaginated wing rudiments in the final larval instar represent an autapomorphy of the Endopterygota groundplan. This interpretation was based on the assumption that Nannochoristidae constitutes a basal lineage within Mecoptera. Beutel and Friedrich (2019), in contrast, proposed that Nannochoristidae represents a lineage distinct from other Mecoptera and should be treated as a separate suborder. Tihelka et al. (2020) conducted a molecular phylogenetic analysis of Mecoptera and found that Nannochoristidae is consistently recovered as sister to Siphonaptera (fleas). Their data did not support the hypothesis that Nannochoristidae is the basal lineage of Mecoptera. The developmental analysis of *Mavropanorpa* presented here highlights the late‐forming, non‐invaginated nature of the wing primordium, contrasting with the deeply invaginated morphology and peripodial sheath observed in *Nannochorista*. Although this does not conclusively show that Nannochorista has an early‐forming condition, the growth of its primordia during the active final instar suggests a derived condition. In contrast, *Mavropanorpa*, as examined in this study, exhibits no such growth during the active final instar, instead showing a late‐forming condition that may be more consistent with the inferred ancestral condition than the condition observed in Nannochoristidae. This contrast is consistent with the hypothesis that the deeply invaginated morphology of *Nannochorista* represents a derived condition, consistent with molecular phylogenies (Tihelka et al. 2020) that place it as a sister to Siphonaptera. However, developmental data on wing primordium formation are currently unavailable for Siphonaptera, the sister group of Nannochoristidae in recent phylogenies; therefore, the evolutionary interpretation of the Nannochorista condition remains provisional. Our findings provide a stage‐specific developmental framework for *M. japonica*, offering a comparative basis for future analyses of wing development in other mecopteran lineages such as Boreidae and Bittacidae. These results not only address a long‐standing gap in our understanding of mecopteran metamorphosis but also have broader implications for interpreting the evolutionary diversification of wing primordium development, particularly among taxa exhibiting late‐forming wing discs.

Further, our study raises the question of whether the underlying genetic program for wing development is initiated before visible morphogenesis. Evidence from other holometabolous insects suggests that this is plausible. In *Tribolium castaneum*, for example, the wing‐patterning gene vestigial (*vg*) is expressed from embryonic stages onward, and *vg* knockdown in the penultimate instar abolishes subsequent disc formation (Clark‐Hachtel et al. 2013). Although disc morphogenesis in *M. japonica* is delayed until the prepupal stage, early activation of wing primordium identity genes cannot be excluded.

The timing and organization of wing development are relevant to discussions on the evolutionary origin of complete metamorphosis and competing models of stage homology among the larva, nymph/pronymph, and pupa (Truman and Riddiford 1999, 2002; Truman 2019). While we do not attempt to resolve these homology models here, our stage‐specific data provide comparative context for evaluating how shifts in developmental timing and morphogenesis relate to broader evolutionary scenarios. Truman (2019) emphasized that the evolution of holometaboly involved heterochronic shifts and endocrine reorganization that redefined the relationships among embryonic, larval, pupal, and adult stages. By documenting a late‐forming, non‐invaginated mode of wing development in a phylogenetically informative mecopteran lineage, the present study provides comparative morphological data that may contribute to understanding how developmental timing was reorganized during the transition to complete metamorphosis. Further comparative and functional analyses will be required to determine how such temporal shifts relate to the evolutionary relationship between larval and nymphal stages.

Recent functional genetic advances further enhance the value of Mecoptera as an evolutionary developmental model. Liu and Hua (2024) demonstrated the feasibility of parental RNA interference in the scorpionfly *Panorpa liui*, successfully knocking down the Hox genes *Ultrabithorax* and *abdominal‐A* and revealing their distinct roles in embryonic abdominal appendage development. This study provides direct evidence that gene function can be experimentally manipulated in scorpionflies, overcoming a long‐standing technical limitation for this order. When combined with the stage‐specific morphological framework of wing primordium development presented here, the availability of RNAi techniques establishes Mecoptera as a promising comparative system for investigating the evolution of imaginal primordia, heterochrony in appendage development, and the developmental basis of complete metamorphosis. Given the phylogenetic position of Mecoptera within Antliophora, scorpionflies are particularly well suited to bridging mechanistic insights between Diptera and other holometabolous lineages.

While the late‐forming condition has been interpreted as potentially representing an ancestral condition of Holometabola in previous studies, our findings raise important considerations. As *M. japonica*, the focal species of this study, does not occupy a clearly basal position within Mecoptera, the late‐forming, non‐invaginated wing primordium observed in this species may represent a lineage‐specific rather than ancestral condition. Comparative ontogenetic analyses across more basal holometabolous lineages will be necessary to resolve the evolutionary history of wing primordium morphogenesis.

## 5 Conclusion

**Figure 3:**
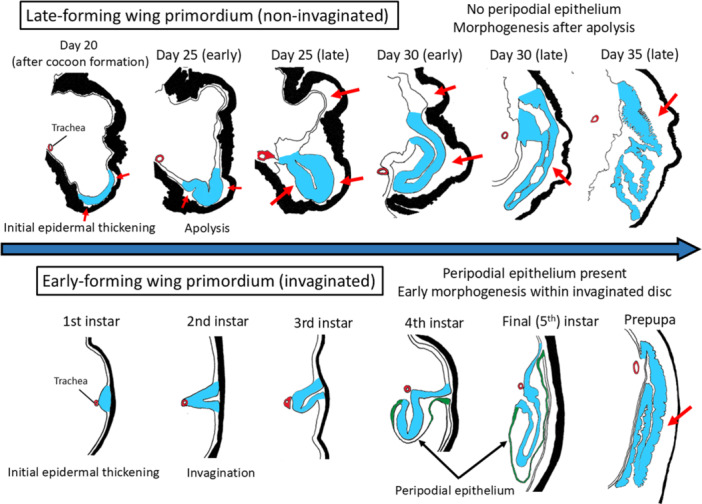
Schematic diagram of wing primordium development in Holometabola. The late‐forming, non‐invaginated condition observed in Mecoptera (*Mavropanorpa japonica*) is contrasted with the early‐forming, invaginated condition characteristic of Lepidoptera (*Papilio xuthus*), modified from Hidaka (1975). In *M. japonica*, wing primordia arise as localized epidermal thickenings concurrent with apolysis and remain continuous with the larval epidermis without forming a morphologically distinct peripodial epithelium. Morphogenesis proceeds externally beneath the larval cuticle. Red arrowheads indicate the region where apolysis occurs. In contrast, in Lepidoptera, wing discs invaginate early, form a morphologically distinct peripodial epithelium, and initiate morphogenesis within an enclosed epithelial pouch. The dorsal surface is at the top. [Color figure can be viewed at wileyonlinelibrary.com]

The present study provides the first stage‐specific description of late‐forming, externally developing wing primordia in Mecoptera. We found that apolysis onset is synchronized with the initial thickening of the wing primordium, constituting a stage‐specific criterion for late‐forming, non‐invaginated primordia, and confirmed the absence of a morphologically distinct peripodial epithelium at any stage examined (Figure 3). These findings provide new insights into the evolutionary diversity of wing morphogenesis in Endopterygota and highlight the importance of broader comparative analyses across both basal and derived mecopteran lineages.

## Conflicts of Interest

The authors declare no conflicts of interest.

## Data Availability

The data that support the findings of this study are available from the corresponding author upon reasonable request.
